# Effects of temporal IFNγ exposure on macrophage phenotype and secretory profile: exploring GMP-Compliant production of a novel subtype of regulatory macrophages (Mreg_IFNγ0_) for potential cell therapeutic applications

**DOI:** 10.1186/s12967-024-05336-y

**Published:** 2024-06-04

**Authors:** Karina Zitta, Lars Hummitzsch, Frank Lichte, Fred Fändrich, Markus Steinfath, Christine Eimer, Sebastian Kapahnke, Matthias Buerger, Katharina Hess, Melanie Rusch, Rene Rusch, Rouven Berndt, Martin Albrecht

**Affiliations:** 1 Department of Anesthesiology and Intensive Care Medicine, University Hospital Schleswig-Holstein (UKSH), Kiel, Germany; 2 Department of Anatomy, University of Kiel, Kiel, Germany; 3 Clinic for Applied Cell Therapy, UKSH, Kiel, Germany; 4 Clinic for Vascular and Endovascular Surgery, UKSH, Kiel, Germany; 5 Department of Pathology, UKSH, Kiel, Germany

**Keywords:** Macrophages, Monocytes, Cell therapy, M1/M2 macrophages

## Abstract

**Background:**

Macrophages are involved in tissue homeostasis, angiogenesis and immunomodulation. Proangiogenic and anti-inflammatory macrophages (regulatory macrophages, Mreg) can be differentiated in-vitro from CD14^+^ monocytes by using a defined cell culture medium and a stimulus of IFNγ.

**Aim of the study:**

To scrutinize the potential impact of temporal IFNγ exposure on macrophage differentiation as such exposure may lead to the emergence of a distinct and novel macrophage subtype.

**Methods:**

Differentiation of human CD14^+^ monocytes to Mreg was performed using a GMP compliant protocol and administration of IFNγ on day 6. Monocytes from the same donor were in parallel differentiated to Mreg_IFNγ0_ using the identical protocol but with administration of IFNγ on day 0. Cell characterization was performed using brightfield microscopy, automated and metabolic cell analysis, transmission electron microscopy, flow cytometry, qPCR and secretome profiling.

**Results:**

Mreg and Mreg_IFNγ0_ showed no differences in cell size and volume. However, phenotypically Mreg_IFNγ0_ exhibited fewer intracellular vesicles/vacuoles but larger pseudopodia-like extensions. Mreg_IFNγ0_ revealed reduced expression of IDO and PD-L1 (P < 0.01 for both). They were positive for CD80, CD14, CD16 and CD38 (P < 0.0001vs. Mreg for all), while the majority of Mreg_IFNγ0_ did not express CD206, CD56, and CD103 on their cell surface (P < 0.01 vs. Mreg for all). In terms of their secretomes, Mreg_IFNγ0_ differed significantly from Mreg. Mreg_IFNγ0_ media exhibited reduced levels of ENA-78, Osteopontin and Serpin E1, while the amounts of MIG (CXCL9) and IP10 were increased.

**Conclusion:**

Exposing CD14^+^ monocytes to an alternatively timed IFNγ stimulation results in a novel macrophage subtype which possess additional M1-like features (Mreg_IFNγ0_). Mreg_IFNγ0_ may therefore have the potential to serve as cellular therapeutics for clinical applications beyond those covered by M2-like Mreg, including immunomodulation and tumor treatment.

**Supplementary Information:**

The online version contains supplementary material available at 10.1186/s12967-024-05336-y.

## Introduction

Macrophages exhibit an extensive subtype diversity which is influenced by tissue-specific cues and local cytokine regulation. The functional diversity of macrophage subtypes encompasses e.g. pro-inflammatory, anti-inflammatory, and angiogenic effects [1, 2]. A comprehensive insight into the mechanisms governing macrophage differentiation and their functions is pivotal for understanding diseases involving macrophages and is also crucial for the future establishment of individualized cell therapeutic approaches in various diseases.

Monocytes can be easily and abundantly extracted from blood of donors or patients [3]. Former studies have demonstrated that these monocytes, through the administration of different cytokines (e.g. IL-4, IL-13, IFNγ), can be differentiated into distinct macrophage subtypes with diverse functions (e.g., M1, M2 macrophages) [2]. One of these macrophage subtypes, the so-called regulatory macrophage (Mreg), has already been successfully employed in clinical studies to reduce rejection reactions and immunosuppressive treatment following kidney transplantation [4–6].

The authors have previously elucidated the production of Mreg capable of suppressing T-cell activation and promoting pro-angiogenic responses [7, 8]. Presently, we are preparing for a clinical phase I/II study employing pro-angiogenic Mreg in treating patients with chronic limb threatening ischemia (CLTI). Therefore, allogeneic Mreg are isolated and differentiated from monocytes under GMP-compliant conditions, involving IFNγ administration on day 6.

In light of recent observations and literature suggesting the inducibility of different macrophage subtypes via various cytokine combinations, the current study explores the possibility of generating a distinct and novel macrophage subtype by temporally altering IFNγ administration while adhering to the established GMP-compliant protocol. The here presented findings indicate that early IFNγ administration during the differentiation process results in a robust macrophage subtype (Mreg_IFNγ0_) that is phenotypically, metabolically, and in terms of CD markers and secretome profile distinct from the established Mreg and may bear the potential for a future cell therapeutic application.

## Methods

### Production of Mreg_IFNγ0_

**Fig. 1:**
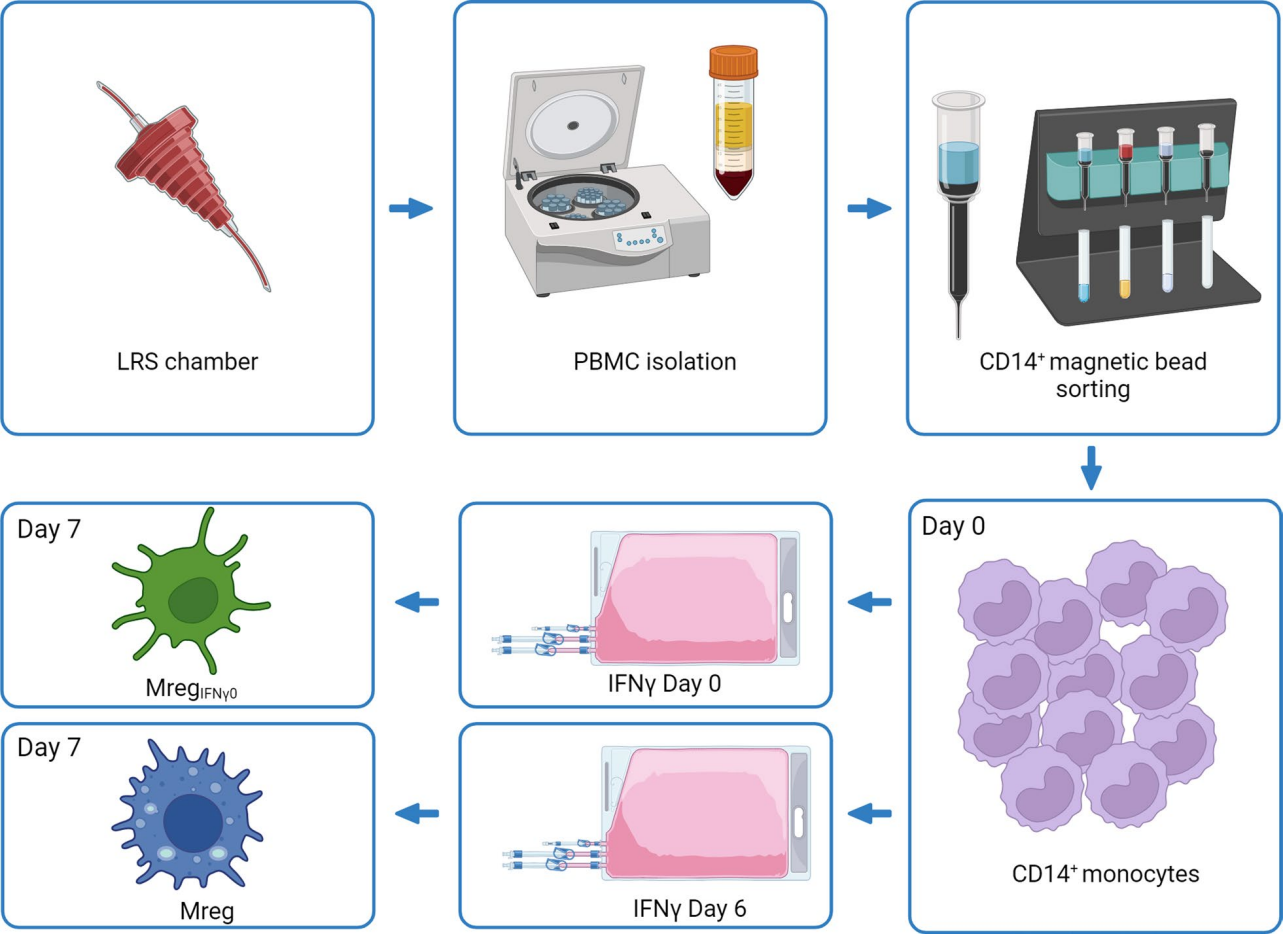
Schematic representation of the manufacturing process of Mreg and Mreg_IFNγ0_. Monocytes are obtained from leukocytes of healthy donors through density centrifugation and CD14 Magnetic Bead Sorting. Subsequently, the monocytes are cultured for 7 days in cell culture bags using a defined differentiation medium, with the addition of IFNγ on Day 0 and Day 6 to obtain Mreg_IFNγ0_ and Mreg_,_ respectively. A detailed protocol is provided as Supplement 1

With a scientific partnership in place, the GMP-compliant manufacture of Mreg for a clinical study (phase I/II trial) involving patients with CLTI is presently ongoing (https://biologics.catalent.com/catalent-news/catalent-signs-development-and-manufacturing-agreement-with-trizell-for-macrophage-based-advanced-cell-therapy/). The exact GMP-compliant manufacturing process is confidential, while the production of Mreg for research purposes is detailed in the protocol provided as Supplement 1. To produce Mreg_IFNγ0_, the identical protocol is used, with the only modification being that the administration of IFNγ is carried out on day 0 instead of day 6. Figure 1 simplifies the manufacturing and differentiation process of Mreg and Mreg_IFNγ0_.

### Measurement of cell viability, cell size, cell volume and metabolic parameters

Automated cell analysis involved the assessment of cell viability, cell size as well as cell volume employing two distinct methods: (i) The MOXI cell counter (Orflo, Ketchum, ID, USA), which uses the Coulter principle to analyze membrane-surrounded structures ranging in size from 3 to 20 µm. (ii) The Nucleocounter (NC-200 Chemometec, Allerod, Denmark), which utilizes two different dyes to stain cell nuclei, facilitating the differentiation between live and dead cells. To assess differences in the metabolic activities between Mreg and Mreg_IFNγ0_, metabolic parameters as pH, glucose concentration and lactate concentration were evaluated in cell culture supernatants on Day 7 of the differentiation period using a blood gas analyser (BGA® Gem premier 3000, Instrumentation Laboratory Company, Bedford, USA).

### Gene expression analyses

Cells underwent two washing steps with phosphate-buffered saline (Sigma-Aldrich) and were then lysed in RLT buffer (Qiagen, Hilden, Germany). RNA isolation was carried out using the RNeasy Minikit following the manufacturer's protocol (Qiagen). Total RNA was utilized to generate cDNA with the High Capacity cDNA Reverse Transcription Kit (Thermofisher Scientific, Vilnius, Lithuania). 1 ng of sample in a final volume of 10 μl served as template for PCR experiments, employing innuMIX qPCR DSGreen (Analytikjena, Jena, Germany). Specific fragments of human transcripts were amplified using the following primers (Metabion, Martinsried, Germany): Indoleamine 2,3-dioxygenase (IDO): Forward 5′-ATGCAGACTGTGTCTTGGCA-3′, Reverse 5′-GCGCCTTTAGCAAAGTGTCC-3′; Programmed Death-Ligand 1 (PD-L1): Forward 5′-ATGGTGGTGCCGACTACAAG**-**3′, Reverse 5′-GGAATTGGTGGTGGTGGTCT-3′; Dehydrogenase/Reductase SDR Family Member 9 (DHRS9): Forward 5′-TGACCGACCCAGAGAATGTCA**-**3′, Reverse 5′-GCCGGGAACACCAGCATTATT-3′. Real time PCR products were generated and visualized with the qTOWER^3^ (Analytikjena). Relative quantifications (RQ) from each gene of interest (GOI) were calculated using the qPCR intensities obtained from each sample (Mreg or Mreg_IFNγ0_) as delta CT (ΔCT = GOI-Housekeeping gene) and relativized to the control (monocyte) qPCR intensities following the formula:

$${\text{RQ}}= 2^{-(\Delta {\text{CT Mreg}}/{\text{MregIFN}}{\upgamma}0 - \Delta {\text{CT monocytes}})}$$

### Flow cytometry

Flow cytometry analysis was conducted using the MACS Q10™ cytometer from Miltenyi. Specific antibodies and their corresponding isotypes, sourced from BD Biosciences, were directly conjugated with fluorescein isothiocyanate (FITC) for CD31, CD16, anti-mouse IgG1κ; with phycoerythrin (PE) for CD80, CD86, CD38, CD11c, anti-mouse IgG1κ; and with allophycocyanin (APC) for CD56, CD206, CD103, anti-mouse IgG1κ, CD14, anti-mouse IgG2a. REA antibodies from Miltenyi directly conjugated with allophycocyanin (APC) were employed for PD-L1 and IgG1 detection. The gating strategy involved (i) identifying the Mreg and Mreg_IFNγ0_ populations based on their size and granularity (FSC/SSC profiles), (ii) excluding non-viable cells (via 7-AAD exclusion, BD Biosciences), (iii) characterizing Mreg and Mreg_IFNγ0_ using the respective CD specific antibodies.

### Transmission electron microscopy

Mreg and Mreg_IFNγ0_ pellets were fixed in 3% glutaraldehyde in PBS for 30 min. Subsequently, post-fixation was performed in 2% osmium tetroxide for 2 × 5 min, followed by dehydration in an ascending series of ethanol. The specimens were then embedded in 1:1 araldite/ethanol for 60 min at room temperature, 40 °C for 3 days and then left at 65 °C overnight. The araldite block was trimmed for ultra-thin sectioning, and ultra-thin sections (40–50 nm) were cut using the Ultramicrotome Leica UC7 with a diamond knife (Diatom, Hatfield, PA, USA). The resulting sections were contrasted with uranyl acetate for 15 min and lead citrate for 7 min. The analysis was conducted using a transmission electron microscope (Jeol JEM1400plus) connected to a digital imaging system (TVIPS TemCam-F416).

### Secretome analyses

The analysis of secreted cytokines was performed using a human proteome profiling array (ARY022B, R&D Systems) following the assay kit protocol provided by the manufacturer with minor modifications. The relative amounts of cytokines were assessed by densitometric analyses of the arrays using ImageJ 1.41 software (NIH). Optical density measurements were taken for each spot on the membrane subtracting the background optical density. The cutoff signal level was established at a value of 10% of the respective reference spots. Secretome profiling analyses were conducted to provide an overview of potential differences in the secretome between Mreg and Mreg_IFNγ0_. Equal amounts of cell culture supernatants (120 µl) from Mreg and, in a parallel approach, equal amounts (120 µl) of cell culture supernatants from Mreg_IFNγ0_ from the same 5 batches were pooled and analyzed. Pooling the respective samples may pose a potential limitation, as any donor- or batch-specific differences that may exist could be masked and not visible in the overall analysis. Due to this limitation and to avoid overinterpretation of the results, the focus of the proteome profiling array analysis was solely placed on the 5 proteins that exhibited the greatest differences in secretion between Mreg and Mreg_IFNγ0_.

### Statistics

Mreg and Mreg_IFNγ0_ batches generated from leukapheresis samples obtained from 13 different healthy donors were utilized. Due to technical constraints and the limited number of cells available per donor, experiments were conducted with differing numbers of Mreg and Mreg_IFNγ0_ batches, ranging from 5 to 13 batches per experiment. However, Mreg and Mreg_IFNγ0_ were differentiated in parallel and each batch originated from the same donor. GraphPad Prism 10.1.0. For Windows (GraphPad Software, San Diego, USA) served as the statistical software for group comparisons. Prior to analysis, all data underwent normality testing using the Kolmogorov–Smirnov test. In instances where normality was not achieved, data were transformed (arcsin of the square root of x) and subjected to one-way ANOVA with Tukey's post-test. A P value < 0.05 was considered statistically significant. Results are presented as mean ± standard error mean (SEM).

## Results

### Phenotypic and metabolic characterization of Mreg_IFNγ0_

**Fig. 2:**
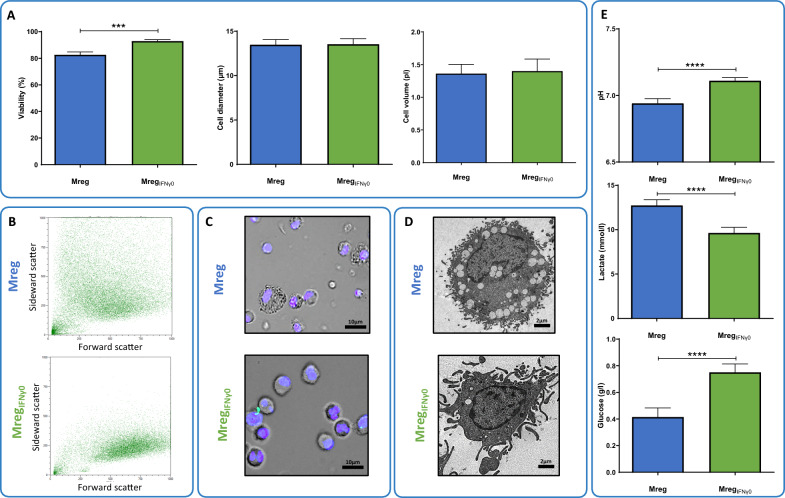
Phenotypic and metabolic characterization of Mreg_IFNγ0_. **A** Determination of cell viability, cell diameter, and cell volume. **B** Estimation of relative cell size and cell granularity using forward and side scatters (FSC, SSC). **C** Brightfield microscopic and **D** Transmission electron microscopic images of Mreg and Mreg_IFNγ0_. **E** Analyses of metabolic parameters employing cell culture supernatants. ***, P < 0.001; ****, P < 0.0001

Both Mreg and Mreg_IFNγ0_ exhibited high viability on day 7 post-harvest (Mreg: 82.68 ± 7.48%, Mreg_IFNγ0_: 92.92 ± 4.12%; P < 0.001), but no significant differences in cell size (Mreg: 13.48 ± 2.09µm, Mreg_IFNγ0_: 13.53 ± 2.29µm; P > 0.05) or cell volume (Mreg: 1.36 ± 0.51pl, Mreg_IFNγ0_: 1.39 ± 0.67pl; P > 0.05; Fig. 2A). Flow cytometric analyses confirmed the similar size distribution between Mreg and Mreg_IFNγ0_ (FSC, Fig. 2B), although Mreg appeared more granulated than Mreg_IFNγ0_ (SSC, Fig. 2B). These results are corroborated by both brightfield microscopic data (Fig. 2C) and electron microscopic analyses: Ultrastructurally, Mreg display numerous intracellular vesicles/vacuoles with a diameter of 0.5–1.0 µm. In contrast, the cytoplasm of Mreg_IFNγ0_ is mainly devoid of vesicular structures, but long pseudopodia-like extensions of the cell membrane are evident (Fig. 2D). Indications of distinct metabolic activities between Mreg and Mreg_IFNγ0_ were observed by analysis of pH, glucose, and lactate in cell culture supernatants on Day 7. Cultures of Mreg_IFNγ0_ exhibited significantly elevated pH values and glucose concentrations compared to Mreg, while lactate levels in the culture medium were decreased (pH: Mreg: 6.94 ± 0.036, Mreg_IFNγ0_: 7.11 ± 0.023; P < 0.0001; Lactate: Mreg: 12.73 ± 0.64 mg/dl, Mreg_IFNγ0_: 9.64 ± 0.63 mg/dl; P < 0.0001; Glucose: Mreg: 0.41 ± 0.07 mg/dl, Mreg_IFNγ0_: 0.75 ± 0.06 mg/dl; P < 0.0001; Fig. 2E).

### Gene expression and cell surface characterization of Mreg_IFNγ0_

**Fig. 3:**
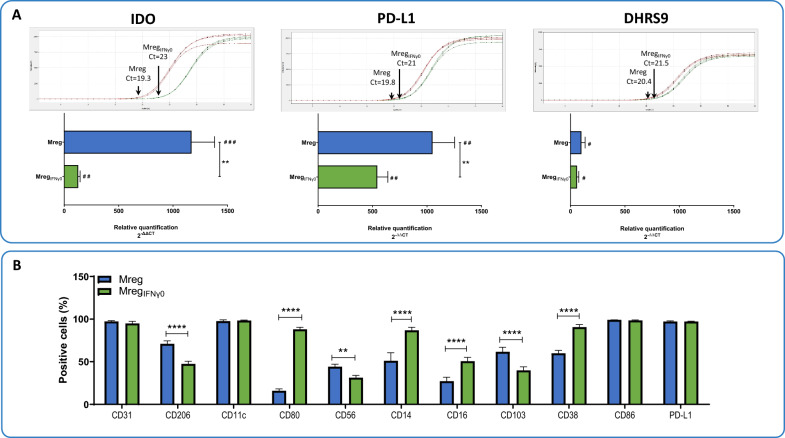
Gene expression and cell surface characterization of Mreg_IFNγ0_. **A** Quantification of the expression of key genes involved in immune cell response and macrophage characterization. Graphs above the columns denote the cycle threshold (Ct) for each cell type. **B** Characterization of Mreg_IFNγ0_ regarding the presence of typical immune cell-associated cell surface molecules. Results are presented as mean ± standard error mean (SEM). **, P < 0.01; ****, P < 0.0001; ^#^, P < 0.05; ^##^, P < 0.01 and.^###^, P < 0.001 vs. monocytes (control)

Indoleamine 2,3-dioxygenase (IDO), Programmed Death - Ligand 1 (PD-L1), and Dehydrogenase/Reductase SDR Family Member 9 (DHRS9) play a crucial role in the context of immune cell modulation and macrophage subtype characterization. Mreg_IFNγ0_, in contrast to Mreg, expressed significantly lower levels of IDO and PD-L1 mRNA [IDO Mreg (relative quantification, RQ): 1170 ± 212.1, IDO Mreg_IFNγ0_ (RQ): 128.8 ± 17.04; P < 0.01; PD-L1 Mreg (RQ): 1051.1 ± 202.8, PD-L1 Mreg_IFNγ0_ (RQ): 543.3 ± 96.97; P < 0.01], while no difference was observed between Mreg_IFNγ0_ and Mreg regarding DHRS9 expression [DHRS9 Mreg (RQ): 99.9 ± 34.24, DHRS9 Mreg_IFNγ0_ (RQ): 60.16 ± 15.56; P > 0.05; Fig. 3A].

Mreg_IFNγ0_ were further analyzed by flow cytometry for the presence of typical macrophage and immune cell-associated CD molecules on the cell surface. Significant differences were observed between Mreg and Mreg_IFNγ0_ in terms of the percentage of cells positive for the following CD markers: CD206: Mreg: 71.03 ± 3.56% and Mreg_IFNγ0_: 47.42 ± 3.24%; P < 0.0001; CD80: Mreg: 15.90 ± 2.27% and Mreg_IFNγ0_: 88.08 ± 2.36%; P < 0.0001; CD56: Mreg: 44.24 ± 2.86% and Mreg_IFNγ0_: 31.34 ± 2.61%; P < 0.01; CD14: Mreg: 51.06 ± 9.44% and Mreg_IFNγ0_: 86.86 ± 3.63%; P < 0.0001; CD16: Mreg: 27.19 ± 4.79% and Mreg_IFNγ0_: 50.68 ± 4.49%; P < 0.0001; CD103: Mreg: 61.64 ± 5.37% and Mreg_IFNγ0_: 39.82 ± 4.25%; P < 0.0001; CD38: Mreg: 59.87 ± 3.55% and Mreg_IFNγ0_: 90.57 ± 3.06%; P < 0.0001; Fig. 3B).

### Characterization of the Mreg_IFNγ0_ secretome

**Fig. 4:**
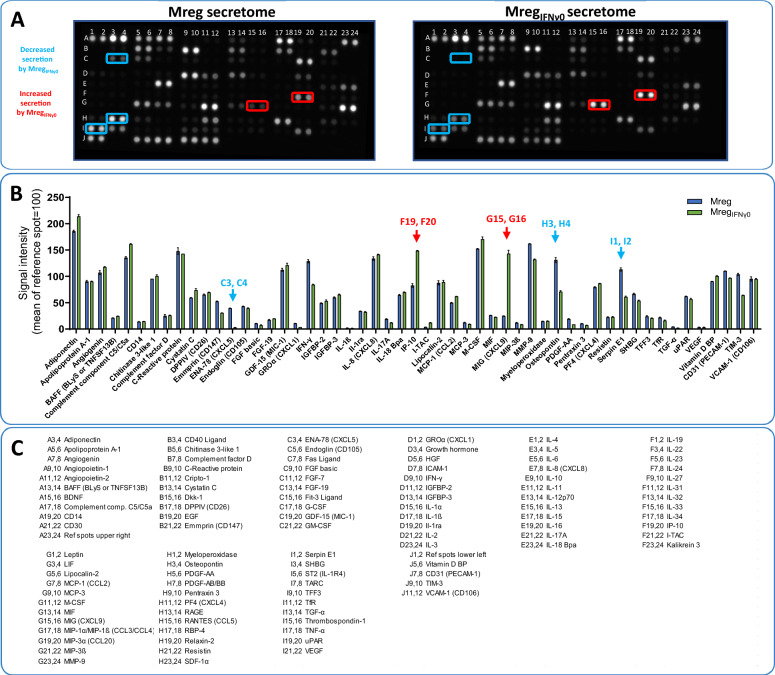
Secretome characterization of Mreg and Mreg_IFNγ0_. **A** Proteome arrays depicting the cytokine-based secretome of Mreg on day 7 (left) and Mreg_IFNγ0_ on day 7 (right). Coordinates indicate cytokine locations, with colored squares highlighting differentially secreted proteins. **B** Graph illustrating the most abundantly secreted cytokines. **C** List of cytokine location on the array membrane. Results are presented as mean ± standard error mean (SEM)

To further investigate the cytokines secreted by Mreg_IFNγ0_, proteome profiling arrays were employed [8, 9]. The results indicate that both Mreg and Mreg_IFNγ0_ release a variety of cytokines involved in immunoregulation. Despite similarities in cytokine secretion patterns, significant differences between Mreg and Mreg_IFNγ0_ were noted regarding the relative amounts of various factors released into the culture medium. Specifically, the secretion of the following cytokines was reduced in Mreg_IFNγ0_: ENA-78 (CXCL5): 2.76 ± 0.22% (Mreg_IFNγ0_) and 39.6 ± 0.37% (Mreg); Osteopontin: 70.98 ± 1.83% (Mreg_IFNγ0_) and 131.12 ± 4.51% (Mreg); Serpin E1: 61.18 ± 1.47% (Mreg_IFNγ0_) and 113.12 ± 2.99% (Mreg; Fig. 4). In contrast, increased levels of the following cytokines were detected in the culture medium of Mreg_IFNγ0_: IP-10 (CXCL10): 148.63 ± 0.75% (Mreg_IFNγ0_) and 82.89 ± 3.46% (Mreg); MIG (CXCL9): 143.30 ± 6.47% (Mreg_IFNγ0_) and 24.93 ± 0.15% (Mreg; Fig. 4).

## Discussion

Macrophages play a pivotal role in host defense and tissue homeostasis. They are known for their ability to phagocytose foreign particles, microbes, and cellular debris, and initiate immune responses by presenting antigens to other immune cells. Macrophages also exhibit tissue-specific functions, participating in processes such as wound healing, inflammation, immune regulation, tumor resistance/progression, tissue remodeling, and possibly angiogenesis [1, 2].

Different research groups have demonstrated in recent years that distinct macrophage subtypes can be differentiated in vitro by the administration of various cytokines/factors (e.g., IL-4, IL-13, IFNγ, LPS), signifying the potential of these in vitro-differentiated cell types in the context of cell therapy [10, 11]. We and others have shown that the administration of IFNγ on day 6 of the differentiation period can generate an anti-inflammatory, T-cell suppressive, and potentially pro-angiogenic macrophage subtype known as regulatory macrophages (Mreg) [7, 8, 12–15]. These Mreg cells have already been successfully employed in a clinical study to reduce rejection reactions in kidney transplantations [4, 6].

Prior research conducted by the authors has been dedicated to characterizing Mreg for several years [8, 12, 16], demonstrating their ability to secrete pro-angiogenic factors, particularly under hypoxic conditions [8]. Building upon these findings, a GMP-compliant protocol for the production of Mreg was developed and a phase I/II study employing Mreg for treating patients with chronic limb threatening ischemia (CLTI) is under preparation.

The present study demonstrates that the temporally adjusted administration of IFNγ at Day 0, while adhering to the GMP-compliant protocol, leads to a cell type (Mreg_IFNγ0_) distinct from the previously described Mreg. Based on our flow cytometric and electron microscopical findings that revealed Mreg_IFNγ0_ as cells with few intracellular vesicles/vacuoles but numerous pseudopodia, Mreg_IFNγ0_ are possibly involved in essential cellular functions such as movement, phagocytosis, chemotaxis, interaction with the extracellular matrix, and sensory perception [17, 18]. Regarding metabolic parameters, we identified an elevated pH, increased glucose concentration, and reduced lactate levels in the culture medium of Mreg_IFNγ0_ on Day 7 of the differentiation period. An increased pH not only enhances antimicrobial activity but also drives polarization towards the classically activated M1 macrophage state, reinforcing their possible involvement in inflammatory responses [19]. On the other hand, culture media from Mreg_IFNγ0_ exhibited high glucose levels, which is untypical for inflammatory cells that rely on glycolysis for energy production [10, 20]. Regarding lactate, recent studies attribute to it a function as a second messenger and it has been demonstrated that lactate can shift macrophage differentiation towards anti-inflammatory M2 macrophages [21, 22]. The observation that culture media from Mreg_IFNγ0_ show significantly lower lactate levels compared to anti-inflammatory Mreg further supports the hypothesis that Mreg_IFNγ0_ represent a novel subtype of macrophages, distinct from the previously described Mreg.

IDO and PD-L1 contribute to the modulation of the immune system, playing a role in its suppression. IDO is involved in mitigating excessive inflammatory responses and fosters the induction of immune tolerance [23, 24]. PD-L1 is instrumental in preserving tissue homeostasis by preventing undesired immune reactions that might result in tissue damage. It ensures a balanced immune activity, enabling appropriate responses to pathogens while preventing excessive inflammation [25]. IDO as well as PD-L1 also inhibit T-cell activation [22, 26, 27]. The significantly lower expression of IDO and PD-L1 by Mreg_IFNγ0_ compared to Mreg suggests a less pronounced T-cell suppressive effect, reaffirming the molecular and likely functional distinctiveness between Mreg and Mreg_IFNγ0_. It is worth mentioning that despite the lower PD-L1 expression in Mreg_IFNγ0_, the same percentage of Mreg and Mreg_IFNγ0_ are positive for PD-L1 on protein level. This observation could be attributed to different post-transcriptional or post-translational processes in Mreg and Mreg_IFNγ0_, or to the possibility that even small amounts of mRNA might be adequate to produce sufficient quantities of PD-L1 protein per cell, so that as a result no differences in the number of PD-L1-positive Mreg and Mreg_IFNγ0_ are revealed by flow cytometry.

The flow cytometric data on the expression of typical immunorelevant CD molecules also suggest that the Mreg_IFNγ0_ population represents a novel macrophage subtype different from the already known Mreg. In contrast to Mreg, which are mostly negative for CD80, the majority of Mreg_IFNγ0_ are positive for this molecule. CD80 interacts with its ligand, CD28 on the surface of T-cells, providing a co-stimulatory signal that enhances T-cell activation and proliferation [28], leading to the production of cytokines and the development of immune responses against pathogens [28]. Similar observations apply to CD14, which is found on the predominant fraction of Mreg_IFNγ0_. CD14 is involved in the recognition of bacterial antigens, facilitating the activation of immune responses by promoting the binding of LPS to Toll-like receptor 4, thereby initiating inflammatory signaling pathways [29]. In contrast to Mreg, CD38 is present on over 80% of Mreg_IFNγ0_. Increased CD38 expression is usually associated with pro-inflammatory macrophage subtypes. CD38 plays a role in immune regulation, including lymphocyte activation, proliferation, and cytokine production, and is implicated in cell adhesion and migration processes [30]. This aligns well with the morphological appearance of multiple pseudopodia on Mreg_IFNγ0_. Finally, the observation that only half of the Mreg_IFNγ0_ population is positive for the mannose receptor CD206 suggests at least some pro-inflammatory M1-like features as CD206 is associated with anti-inflammatory functions and is often expressed on macrophages with an alternatively activated, anti-inflammatory phenotype (M2 macrophages) [31].

Regarding their secretory potential, Mreg_IFNγ0_ exhibit several released cytokines that distinctly differ from those of Mreg. These differences in the secretome of Mreg and Mreg_IFNγ0_ further support the previously suggested hypothesis that Mreg_IFNγ0_ represents a new subtype of Mreg. While Mreg_IFNγ0_ share some characteristics with Mreg, they markedly differ in many other features. As examples from the secretome analyses, the two factors MIG (CXCL9) and IP10 (CXCL10) are worth mentioning here. These cytokines are released in large quantities into the culture medium by Mreg_IFNγ0_ but not Mreg. MIG and IP10 serve as chemokines that play pivotal roles in orchestrating immune responses. They act by binding to their respective receptors on immune cells, such as T-cells and natural killer cells, inducing chemotaxis and migration to sites of inflammation. Additionally, they contribute to the regulation of adaptive and innate immunity by modulating the activation and function of various immune cell subsets. MIG and IP10 are also involved in immune surveillance, promoting the clearance of infected or malignant cells [32, 33].

The in-vitro data presented here highlight differences between Mreg and Mreg_IFNγ0_ in terms of phenotype, cell surface receptor composition, metabolic activity, and secretome. Unlike Mreg, which exhibit several M2 typical characteristics, Mreg_IFNγ0_ additionally demonstrate characteristics of pro-inflammatory cell types. Whether this holds true under in-vivo conditions requires clarification through further animal experiments. However, based on the characteristics of Mreg_IFNγ0_ described in this study and data from current pilot experiments, it is plausible that Mreg_IFNγ0_ possesses a certain degree of cellular plasticity, enabling them to induce inflammatory or anti-inflammatory mechanisms depending on the microenvironment. Preliminary data indicate that Mreg_IFNγ0_, similar to Mreg, respond to hypoxic conditions, suggesting that they may mediate different effects based on the locally occurring oxygen concentration. If these assumptions and hypotheses can be confirmed in further in-vivo experiments, a wide range of clinical applications for Mreg_IFNγ0_ would emerge. For instance, systemic administration of Mreg_IFNγ0_ via the bloodstream could allow direct interaction with circulating immune cells, regulating their functions. Local injection of Mreg_IFNγ0_ into hypoxic tumor areas could influence the tumor microenvironment and topical application of Mreg_IFNγ0_ on open wounds could guide and regulate wound healing processes.

In summary, we have demonstrated that the temporal modification of IFNγ administration within an established GMP-compliant protocol for producing anti-inflammatory regulatory macrophages (Mreg) leads to the generation of a novel macrophage subtype which possess additional M1-like features (Mreg_IFNγ0_). These Mreg_IFNγ0_ cells can be easily and reproducibly manufactured from CD14^+^ monocytes based on the established protocol. Mreg_IFNγ0_ may therefore have the potential to serve as cellular therapeutics for clinical applications beyond those covered by M2-like Mreg, including immunomodulation and tumor treatment.

### Supplementary Information

Supplementary Material 1.

## Data Availability

The datasets used and/or analysed during the current study are available from the corresponding author on reasonable request.
