# Supplementary material for: Effects of temporal IFNγ exposure on macrophage phenotype and secretory profile: exploring GMP-Compliant production of a novel subtype of regulatory macrophages (MregIFNγ0) for potential cell therapeutic applications
Source: J Transl Med. 2024 Jun 4;22:534. doi: 10.1186/s12967-024-05336-y (PMC11151567; doi:10.1186/s12967-024-05336-y)
Supplement: Supplementary file 1 — Supplementary Material 1. [file 12967_2024_5336_MOESM1_ESM.docx]

**Standard Manufacturing Protocol**

**Regulatory Macrophages**

**Mreg**

-For research purpose only-

| **TABLE OF CONTENTS** | | | | |  |
| --- | --- | --- | --- | --- | --- |
| **1.** | | | **REAGENTS AND MATERIALS** | |  |
|  |  |  |  |  |  |
| **2.** | | | **PREPARATION OF REAGENTS** | |  |
| **2.1** | | | Preparation and storage of M-CSF | |  |
| **2.2** | | | Preparation and storage of IFN-y | |  |
| **2.3** | | | Heat-inactivation and storage of human AB serum | |  |
| **2.4** | | | Preparation of MACS buffer | |  |
| **2.5** | | | Preparation of Mreg culture medium | |  |
| **3.** | | | **STARTING MATERIAL** | |  |
| **3.1** | | | Leukapheresis product | |  |
| **3.2** | | | LRS chambers and typical composition | |  |
| **3.3** | | | Separation of PBMC by Ficoll density gradient centrifugation | |  |
|  | | |  | |  |
| **4.** | | | **MREG CULTURE** | |  |
| **4.1** | | | Day_0 of culture: Isolation of monocytes and seeding culture bags | |  |
| **4.1.1** | | | Isolation of CD14 monocytes by MACS selection | |  |
| **4.1.2** | | | Seeding of Mreg cultures | |  |
| **4.2** | | | Day_1 of culture: Flipping culture bags | |  |
| **4.3** | | | Day_6 of culture: IFN-y stimulation and flipping culture bags | |  |
| **4.4** | | | Day_7 of culture: Cell harvest | |  |
| **5.** | | | **PRODUCT QUALITY CONTROLS** | |  |
| **5.1** | | Extracellular staining of monocytes and Mreg cells | | |  |
|  | |  | | |  |
|  | |  | | |  |
|  | |  | | |  |
|  | | |  | |  |

| **Abbreviation** | **Definition** |
| --- | --- |
| Mreg | Regulatory macrophage |
| DPBS | Dulbecco's modified phosphate buffered saline |
| hrM-CSF | Human recombinant monocyte colony-stimulating factor |
| hrIFN-γ | Human recombinant interferon-gamma |
| LFH | Laminar flow hood |
| PR | Phenol red |
| CPDA | Citrate-Phosphate-Dextrose-Adenine buffer |
| LRS | Leukocyte Reduction System |
|  |  |

**1. REAGENTS AND MATERIALS**

| **Reagent** | **Cat. #** | **Supplier** |
| --- | --- | --- |
| RPMI 1640 with Glutamine | 61870-010 | Gibco-life |
| CD14 microbeads | 130-050-201 | Miltenyi Biotec |
| Human Serum Albumin 25% | 68982-0643-02 | Octapharma (Nova Biologicals) |
| CliniMACS PBS/EDTA buffer 3x1 liter | 200-070-025 | Miltenyi Biotec |
| Dulbecco’s Phosphate Buffered Saline without Ca2+ or Mg2+ 10x | 70011036 | Gibco-life |
| DPBS | 17-512F | Lonza |
| Human AB-Serum | 535-HI | Access Biologicals |
| Recombinant human M-CSF | 216-GMP-025 | R&D |
| Recombinant human Interferon-γ | 285-GMP-100 | R&D |
| FITC Mouse IgG1,k Isotype Control | 555748 | BD |
| FITC Mouse-Anti human CD31 | 555445 | BD |
| PE Mouse IgG1,k Isotype Control | 555749 | BD |
| PE Mouse-Anti human CD11c | 555392 | BD |
| PE Mouse-Anit human CD 86 | 555658 | BD |
| APC Mouse IgG1,k Isotype Control | 555751 | BD |
| APC Mouse-Anti human CD103 | 563883 | BD |
| APC Mouse-Anti human CD206 | 550889 | BD |
| 7-AAD | 559925 | BD |

| **Materials** | **Cat. #** | **Supplier** |
| --- | --- | --- |
| SteriCup+ Steritop 250ml | SCGPUO2RE | Millipore |
| Filtropur BT 250ml | 83.1822.101 | Sarstedt |
| MACS Separation Unit | 130-042-302 | Miltenyi Biotec |
| MACS Multi Stand | 130-042-303 | Miltenyi Biotec |
| LS Columns | 130-042-401 | Miltenyi Biotec |
| MACS GMP Cell Differentiation Bag 100 | 170-076-400 | Miltenyi Biotec |
| MACS GMP Cell Differentiation Bag 500 | 170-076-402 | Miltenyi Biotec |
| BD Perfusion 20ml syringes | REF300296 | BD |
| BD Perfusion 50ml syringes | REF300136 | BD |
| Laminar flow hood Hera Safe | KS12 | Heraeus |
| Medingen WB5 waterbath |  | Medingen |
| Centrifuge Multifuge 3 S-R |  | Heraeus |
| Eppendorf Centrifuge 5702 R |  | Eppendorf |
| Centrifuge Megafuge1.0 |  | Heraeus |
| Blood analyzer Horiba Micros 60 |  | Horiba |
| Pipettus |  | Brandt |
| Pipettus |  | Hirschmann |
| Moxi Z Cellcounter |  | Orflo |
| Blood analyse maschine ABX Micros60 |  | Axonlab |
| Vortex Genie2 |  | Scientific Industries |
| MACSQuant Analyzer 10 |  | Miltenyi Biotec |
| Incubator Hera Cell 150 |  | Heraeus |
| Microscope Leica DMIL |  | Leica |
| Pipettes Eppendorf Reference variable 0,5-10µl |  | Eppendorf |
| Pipettes Eppendorf Reference variable 10-100µl |  | Eppendorf |
| Pipettes Eppendorf Reference variable 100-1000µl |  | Eppendorf |
| Reaction tubes 50ml | 62.547.254 | Sarstedt |
| Reaction tubes 15ml | 62.554.002 | Sarstedt |
| Reaction tubes 15ml | 188261 | Greiner bio-one |
| Reaction tubes 5ml | 55.476 | Sarstedt |
| Serological pipettes 25ml | 86.1685.001 | Sarstedt |
| Serological pipettes 25ml | 760180 | Greiner bio-one |
| Serological pipettes 10ml | 86.1254.001 | Sarstedt |
| Serological pipettes 5ml | 86.1253.001 | Sarstedt |
| Serological pipettes 5ml | 606180 | Greiner bio-one |
| Reaction tube 1,5ml | 72.706.200 | Sarstedt |
| Pipette tips 200µl | 739290 | Greiner |
| Pipette tips 200µl | 70.760.002 | Sarstedt |
| Pipette tips 1000µl | 70.762 | Sarstedt |
| Pipette tips 10µl | 70.1115 | Sarstedt |
| Pipette tips 10µl | 732002 | Brandt |
| Ficoll Separating Solution | L6115 | Biochrome GmbH |

**2. PREPARATION OF REAGENTS**

**2.1 Preparation and storage of rhMCSF** (final conc. in culture medium 25ng/ml)

| **Step** | **Action** | **Check** |
| --- | --- | --- |
| 1 | In a 15 ml Falcon tube, prepare 6ml of a 0.1% solution of human serum albumin in RPMI 1640 |  |
| 2 | Remove vial of 25µg rhM-CSF from -20°C storage. Allow 5 min to equilibrate to RT |  |
| 3 | Transfer a total of 2.5 ml of the solution from step (1) into the rhM-CSF vial in fractions to dissolve and wash-out all protein from the vial |  |
| 4 | Transfer the rhM-CSF solution into a 15 ml Falcon tube |  |
| 5 | Add 2.5ml of human albumin solution from step (1). Mix gently |  |
| 6 | Make 250µl aliquots (each containing 5µg/ml rhMCSF) |  |
| 7 | Store at -80°C or at -20°C for up to 3 months |  |

**2.2 Preparation and storage of rhIFN-γ** (final conc. in culture medium: 25ng/ml)

| **Step** | **Action** | **Check** |
| --- | --- | --- |
| 1 | In a 15 ml Falcon tube, prepare 11ml of a 0.1% solution of human serum albumin in RPMI 1640 medium |  |
| 2 | Remove vial of 100µg rhIFN-γ from -20°C storage. Allow 5 min to equilibrate to RT, centrifuge briefly at 20.000xg |  |
| 3 | Transfer a total of 2.5 ml of the solution from step (1) into the rhIFN-γ vial in fractions to wash-out all protein from the vial and transfer into a 15 ml Falcon tube |  |
| 4 | Add 7.5ml of human albumin solution from step (1). Mix gently |  |
| 5 | Make 250µl aliquots (each containing 25µg/ml rhIFN-γ ) |  |
| 6 | Store at -80°C or at -20°C for up to 3 months |  |

**2.3 Heat inactivation and storage of human AB serum** (final conc. in culture medium: 10%)

| **Step** | **Action** | **Check** |
| --- | --- | --- |
| 1 | Thaw 100ml aliquot of serum at 4°C overnight (from -80°C) (Serum is already heat inactivated) |  |
| 2 | Decontaminate and transfer to a LFH |  |
| 3 | Make 5, 10 or 25ml aliquots of serum in Falcon tubes |  |
| 4 | Store aliquots at -20°C for up to 4 weeks |  |

**2.4 Preparation of MACS buffer**

To produce 100 ml medium. Scale as required.

| **Step** | **Action** | **Check** |
| --- | --- | --- |
| 1 | In LFH transfer 98 ml CliniMACS PBS/EDTA Buffer into a 100ml sterile cup device using a serological pipette |  |
| 2 | Transfer 2.0 ml of 25% human serum albumin to sterile cup |  |
| 3 | Filter mixture. Store at 4°C |  |

**2.6 Preparation of Mreg culture medium**

To produce 100 ml medium. Scale as required.

| **Step** | **Action** | **Check** |
| --- | --- | --- |
| 1 | In LFH transfer 90 ml RPMI-1640 into a 100ml sterile cup using serological pipette |  |
| 2 | Transfer 10ml heat-inactivated hABS into sterile cup using serological pipette |  |
| 3 | Filter medium mixture. Store at 4°C up to 2 weeks |  |

**2.7 Preparation of FACS buffer**

| **Step** | **Action** | **Check** |
| --- | --- | --- |
| 1 | Make stock solution 10% NaN_3_ in DPBS (1x concentration) |  |
| 2 | Transfer 497m1 PBS to a Schott bottle |  |
| 3 | Add 1ml 10% NaN_3_ solution to give an end concentration of 0.02% |  |
| 4 | Add 2ml 0.5M EDTA to give an end concentration of 2mM |  |
| 5 | Add 5g Bovine Serum Albumin (BSA) and allow to dissolve |  |
| 6 | Store solution at 4°C |  |

**3. STARTING MATERIAL**

**3.1 Leukapheresis product**

Leukapheresis is a procedure which separates human leukocytes from circulating blood; erythrocytes and blood plasma are returned.

**3.2 LRS chambers and typical composition**

| **Obtaining apheresate from LRS chamber: —10 min** | | |
| --- | --- | --- |
| **Step** | **Action** | **Check** |
| 1 | Decontaminate outside of LRS chamber using alcohol wash. Transfer to LFH |  |
| 2 | Hold the LRS chamber with the conical point downwards. Clamp the lower tube with sterile surgical forceps |  |
| 3 | Cut the lower tube with sterile scissors below the clamp |  |
| 4 | Cut the upper tube using sterile scissors |  |
| 5 | Hold the LRS chamber over an open 50ml Falcon tube and remove the clamp. |  |
| 6 | Allow the contents of the LRS chamber to run into the Falcon tube |  |

**3.3 Separation of PBMC by density gradient centrifugation**

| **Separation of PBMC by Ficoll density gradient centrifugation: —90 min** | | |
| --- | --- | --- |
| **Step** | **Action** | **Check** |
| 1 | Dilute contents of LRS chamber with 40ml sterile DBPS without Ca ^2+^ or Mg ^2+^ at RT |  |
| 2 | Perform cell count |  |
| 3 | Transfer 20ml Ficoll solution each into two 50ml Falcon tubes at RT |  |
| 4 | Gently overlay diluted apheresate onto Ficoll using a serological pipette |  |
| 5 | Place the layered columns into a swing-bucket centrifuge |  |
| 6 | Centrifuge at RT for 20min at 863g with low acceleration and no brake |  |
| 7 | Remove tubes from centrifuge. Decontaminate and transfer to LFH |  |
| 8 | Recover mononuclear cells from interface using a 10ml serological pipette |  |
| 9 | Transfer mononuclear cells to a 50ml Falcon tube |  |
| 10 | Resuspend cells to 50ml DBPS without Ca ^2+^ or Mg ^2+^ at RT |  |
| 11 | Pellet cells by centrifugation at 550g for 10min at RT |  |
| 12 | Aspirate supernatant and discard |  |
| 13 | Resuspend cell pellet in 50ml DBPS without Ca ^2+^ or Mg ^2+^ at RT |  |
| 14 | Pellet cells by centrifugation at 216g for 10min at RT |  |
| 15 | Aspirate supernatant and discard |  |
| 16 | Resuspend cell pellet in 50ml DPBS without Ca ^2+^ or Mg ^2+^ at RT |  |
| 17 | Pellet cells by centrifugation at 216g for 10min at RT |  |
| 18 | Aspirate supernatant and discard |  |
| 19 | Resuspend cell pellet in 50ml DPBS without Ca^2+^ or Mg^2+^ at RT |  |
| 20 | Pellet cells by centrifugation at 216g for 10min at RT |  |
| 20 | Aspirate supernatant and discard |  |
| 21 | Resuspend cell pellet in 20ml DPBS without Ca^2+^ or Mg^2+^ at RT |  |
| 22 | Count cells using blood analyzer Horiba Micros 60. Also evaluate concentration of monocytes (This information is important for steps in 4.1.1) |  |

**4. MREG CULTURE**

**4.1 Day_0 of culture: Isolation of monocytes and seeding of cultures**

| **4.1.1 Isolation of CD14 monocytes by MACS selection: —90 min** | | |
| --- | --- | --- |
| **Step** | **Action** | **Check** |
| 1 | From the mononuclear cell preparation (see 3.3) take the required volume of PBMC suspension |  |
| 2 | Transfer to 15ml Falcon(up to 10^8^ monocytes/Falcon). Pellet cells at 300g for 6min |  |
| 3 | Resuspend cells in MACS buffer: 80µl per 10^7^ monocytes |  |
| 4 | Add 20µl CD14 reagent per 10^7^ monocytes. Mix gently |  |
| 5 | Incubate for 15min at 4°C |  |
| 6 | Add 5ml MACS buffer each to sample. Mix gently |  |
| 7 | Centrifuge for 6min at 300g |  |
| 8 | Meanwhile place an LS column (one column/10^8^ monocytes) into magnet. Wash column with 3ml cold MACS buffer |  |
| 9 | Aspirate supernatant |  |
| 10 | Resuspend cells in 1ml cold MACS buffer per cup |  |
| 11 | Pass cell suspension through the column (one column per 10^8^ monocytes) |  |
| 12 | Wash cup from step 10 with 1ml of cold MACS buffer (per column) and transfer 1ml to each column |  |
| 13 | Always let suspension completely flow through the column |  |
| 14 | Wash column with 3ml cold MACS buffer 3 times |  |
| 15 | Remove column from magnet and transfer into a 15ml Falcon. Apply 5ml cold MACS buffer |  |
| 16 | Insert column plunger and rapidly eject column contents into the 15ml Falcon |  |
| 17 | Centrifuge for 10min at 300g, resuspend pellet in 2.5 ml Mreg culture medium (per column) |  |
| 18 | Take aliquots of cells for cell count and analysis by FACS (if required) |  |

| **4.1.2 Seeding of Mreg cultures: —30 min** | | |
| --- | --- | --- |
| **Step** | **Action** | **Check** |
| 1 | Prepare the required volume of CD14 monocyte suspension for seeding in a 50ml Falcon |  |
| 2 | For each 100ml bag prepare 21 x 10^6^ cells in 20ml total volume of Mreg culture medium. Add 100µl of rhMCSF stock solution to obtain a final concentration of 25ng/ml rhMCSF  For each 500ml bag prepare 41 x 10^6^ cells in 50ml total volume of Mreg culture medium. Add 250µl of rhMCSF stock solution to obtain a final concentration of 25ng/ml rhMCSF (rhMCSF containing medium has to be prepared freshly) |  |
| 3 | In LFH, remove plunger from 20ml(or 50ml) syringe. Attach syringe to culture bag via luer |  |
| 4 | Fix syringe in upright position |  |
| 5 | Transfer monocyte suspension into syringe and carefully into culture bags:  20ml cell suspension / 100ml bag or 50ml cell suspension / 500ml bag |  |
| 6 | Expel air bubbles from bag. Seal bag. Label bag. Distribute cell suspension around bag. |  |
| 7 | Place bags into 37°C 5% CO_2_ incubator on horizontal shelves for 24 hours |  |

**4.2 Day_1 of culture: Bag flipping**

| **Flipping bag cultures: —5 min** | | |
| --- | --- | --- |
| **Step** | **Action** | **Check** |
| 1 | Remove culture bags from incubator |  |
| 2 | Invert bags |  |
| 3 | Replace culture bags in incubator in inverted position until day 6 |  |

**4.3 Day_6 of culture: IFN-γ stimulation and bag flipping**

| **IFN-γ stimulation and flipping bag cultures: —30 min** | | |
| --- | --- | --- |
| **Step** | **Action** | **Check** |
| 1 | Thaw aliquots of rhIFN-γ as required |  |
| 2 | Remove culture bags from incubator. Transfer to LFH |  |
| 3 | Load required volume of rhIFN-γ stock solution into syringe (i.e. 50µl rhIFN-γ stock solution / 20ml cell suspension / 100ml bag to obtain a final concentration of 25µg/ml) |  |
| 4 | Apply rhIFN-γ to culture bag by injection through rubber membrane. Rinse syringe several times |  |
| 5 | Gently mix by swirling |  |
| 6 | Replace culture bag into incubator in inverted position (i.e. same position as day 0) |  |
| 7 | Incubate for 18 to 24h |  |

**4.4 Day_7 of culture: Cell harvest**

| **Harvesting of Mreg cultures: —40 min** | | |  |
| --- | --- | --- | --- |
| **Step** | **Action** | **Check** |  |
| 1 | Remove culture bags from incubator |  |  |
| 2 | Submerge culture bags in crushed ice for 15 min |  |  |
| 3 | Detach adherent cells by forcefully pulling the bag down over the edge of a flat surface (for details please refer to video documentation!) |  |  |
| 4 | Transfer bags to LFH |  |  |
| 5 | Attach a 20ml (or 50ml) syringe via luer lock to culture bag. Inject 1x the volume of the syringe of air into bag |  | |
| 6 | Shake bags vigorously |  | |
| 7 | Aspirate medium containing Mreg cells. Remove syringe |  | |
| 8 | Transfer syringe content to a 50ml Falcon tube. Remove plunger. |  | |
| 9 | Attach syringe via luer lock to culture bag and add cold DPBS without Ca^2+^ or Mg^2+^ to the bag (i.e. 20ml/100ml bag). Replace plunger. |  | |
| 10 | Shake bags vigorously |  | |
| 11 | Aspirate DPBS without Ca^2+^ or Mg^2+^ containing Mreg cells. Remove syringe |  | |
| 12 | Transfer syringe content to a 50 ml Falcon tube |  | |
| 13 | Centrifuge cell suspension at 300g for 10min |  | |
| 14 | Aspirate supernatant. Resuspend cells in required volume of required medium/buffer/solution culture medium |  | |
| 15 | Perform cell count and FACS analysis: viability by 7-AAD; release marker CD11c, CD206, CD31, CD86 and (optional) patent markers |  | |

**5. PRODUCT QUALITY CONTROLS**

**5.1 Extracellular staining of Mreg cells**

| **Standard extracellular staining of Mreg cells** | | |
| --- | --- | --- |
| **Step** | **Action** | **Check** |
| 1 | Transfer cell suspension from step 4.4 into a 1.5 or 5ml reaction tube. 5 x 10^5^ Mregs are required for each staining reaction. |  |
| 2 | Pellet cells by centrifugation at 550g for 10min |  |
| 3 | Aspirate supernatant |  |
| 4 | Resuspend cells with DBPS maintaining the minimum cell concentration required per reaction tube (~5 x 10^5^  / 100µl reaction tubes) |  |
| 5 | Transfer 100µl of cell suspension into each reaction tubes |  |
| 6 | Add the respective antibody solution. For all antibodies except CD103: 5µl antibody solution / 100µl of cell suspension (CD103: 2.5µl antibody solution / 100µl of cell suspension) |  |
| 7 | Incubate at 4°C in dark for 20min |  |
| 8 | Add 5µl 7-AAD per reaction tube. Incubate at 4°C in dark for 10min |  |
| 9 | Add 2ml DPBS per tube. Vortex |  |
| 10 | Pellet cells by centrifugation at 300g for 6min |  |
| 11 | Aspirate supernatant |  |
| 12 | Resuspend cells in 200µl DPBS for analysis by flow cytometry (MACS Quant Analyzer) |  |
